# Profiling Patients’ Healthcare Needs to Support Integrated, Person-Centered Models for Long-Term Disease Management (Profile): Research Design

**DOI:** 10.5334/ijic.2208

**Published:** 2016-04-29

**Authors:** Arianne MJ Elissen, Dorijn FL Hertroijs, Nicolaas C Schaper, Hubertus JM Vrijhoef, Dirk Ruwaard

**Affiliations:** Department of Health Services Research, CAPHRI School for Public Health and Primary Care, Faculty of Health, Medicine and Life Sciences, Maastricht University, Duboisdomein 30, PO Box 616, 6200MD, Maastricht, the Netherlands; Department of Internal Medicine, CAPHRI School for Public Health and Primary Care, Faculty of Health, Medicine and Life Sciences, Maastricht University Medical Centre, PO Box 5800, 6202AZ, Maastricht, the Netherlands; Department of Patient and Care, Maastricht University Medical Centre, Maastricht, The Netherlands & Saw Swee Hock School of Public Health, National University of Singapore, National University Health System, 1E Kent Ridge Road, Singapore 119228, Singapore

**Keywords:** type 2 diabetes, disease management, tailored care, patient profiles, Triple Aim, study design

## Abstract

**Background:**

This article presents the design of PROFILe, a study investigating which (bio)medical and non-(bio)medical patient characteristics should guide more tailored chronic care. Based on this insight, the project aims to develop and validate ‘patient profiles’ that can be used in practice to determine optimal treatment strategies for subgroups of chronically ill with similar healthcare needs and preferences.

**Methods/Design:**

PROFILe is a practice-based research comprising four phases. The project focuses on patients with type 2 diabetes. During the first study phase, patient profiles are drafted based on a systematic literature research, latent class growth modeling, and expert collaboration. In phase 2, the profiles are validated from a clinical, patient-related and statistical perspective. Phase 3 involves a discrete choice experiment to gain insight into the patient preferences that exist per profile. In phase 4, the results from all analyses are integrated and recommendations formulated on which patient characteristics should guide tailored chronic care.

**Discussion:**

PROFILe is an innovative study which uses a uniquely holistic approach to assess the healthcare needs and preferences of chronically ill. The patient profiles resulting from this project must be tested in practice to investigate the effects of tailored management on patient experience, population health and costs.

## Background

One of the greatest challenges for health systems and economic and social development in Europe is the rising burden of chronic disease [1]. Around 32 percent of Europeans is now chronically ill, with many – especially elderly – people suffering from multiple conditions at the same time [2]. Without action, the chronic disease epidemic in the region will continue to develop rapidly: diabetes prevalence, for example, is expected to increase by 12.6 million cases over the next 15 years [3]. Chronic conditions cause serious disability, lower quality of life and early mortality, and already consume 70 to 80 percent of healthcare budgets across Europe [1].

When it comes to managing chronic disease, thus far the trend in most countries is to treat conditions separately through multidisciplinary care teams using disease-specific guidelines [4]. While such one-dimensional disease management can lead to improved care quality and outcomes [5,6,7,8], its value is quickly decreasing in proportion to rising multimorbidity. For the growing group of patients living with a complex of (interrelated) chronic conditions – such as diabetes, cardiovascular disease, asthma and dementia – disease management means having several care teams working according to different guidelines [10]. This may lead to fragmented care, loss of responsibility among providers, and confusion or even harm for patients [9]. Recent studies of chronic care in Europe also point to overstandardised service provision, limited preventive action, and a lack of support for patients’ self-management [4,10,11]. Overall, the return on investment in chronic disease management seems relatively poor: real improvements in population health are not always achieved and many patients remain dissatisfied about their care, while costs reach unprecedented levels [1,12].

In recent years, there is increasing consensus that better management of chronic conditions requires an approach centered on patients instead of on their primary diagnosed disease [10]. It has become clear that active participation and commitment of patients is critical for achieving any kind of chronic disease control. Hence, their personal healthcare needs and preferences must be taken into account in clinical decision-making. Such individualisation of care, while important for all chronically ill, is particularly relevant for people with type 2 diabetes [13]. Besides generally being considered the ‘quintessential self-managed disease’, type 2 diabetes is a highly heterogeneous condition both in pathogenesis and clinical manifestation [10]. This means that the ‘typical’ diabetes patient does not exist and standardised management is likely to yield differential treatment effects. Indeed, recent research in Germany and the Netherlands shows that unstable, high-risk diabetes patients benefit significantly more from disease management than patients with better disease control for whom such intensive treatment may have little added value [14,15]. Similarly, various large-scale international studies suggest that not all diabetes patients profit from intensive glucose- or blood pressure-lowering therapy, pointing towards characteristics like age, disease duration, comorbidities, and patient attitude as possible effect modifiers [10,13].

Taking into account patient characteristics – with the potential to modify treatment outcomes in chronically ill – in clinical decision-making is important to enable the *right care* to be provided to the *right person* at the *right time*, with a focus on increased patient engagement, self-management and, ultimately, cost containment. However, thus far, it remains unclear which patient features should guide a more tailored approach to chronic care management and how these can be translated into a feasible tool to support professionals and patients in daily practice. This paper describes the design of a three-year, multiple-phase research project entitled ‘*PROFiling patients’ healthcare needs to support Integrated, person-centered models for Long-term disease management (PROFILe)*’, which seeks to fill this significant gap in knowledge and, in so doing, support more patient-centered, sustainable chronic care management in practice.

## Research aims and questions

The PROFILe project aims to develop and validate a novel, practical instrument – in the form of patient profiles – that supports more tailored chronic care management in practice. Unique about the profiles to be developed is that they will combine (bio)medical and non-(bio)medical patient characteristics relevant for determining an optimal treatment strategy for subgroups of patients with similar care needs and preferences. The objective here is not to create a complex network of detailed patient features, but rather to identify a limited number of key characteristics that, when combined into profiles, can serve as an instrument to help tailor the general stipulations of chronic care standards and guidelines in a patient-driven manner. More specifically, the PROFILe project will answer the following research questions:

1. Which (bio)medical and non-(bio)medical patient characteristics are (clinically) relevant for guiding tailored chronic care management?
2. How can those characteristics be combined into a scientifically robust and practicably feasible set of patient profiles?
3. What are patients’ preferences for specific configurations of professional-led care and self-management support per developed patient profile?

Although the objective of PROFILe is explicitly not to develop another disease-specific approach to chronic care management, type 2 diabetes (as primary diagnosis) is used as a starting point for profile development.

## Methods/Design

### Study design

**Figure 1:**
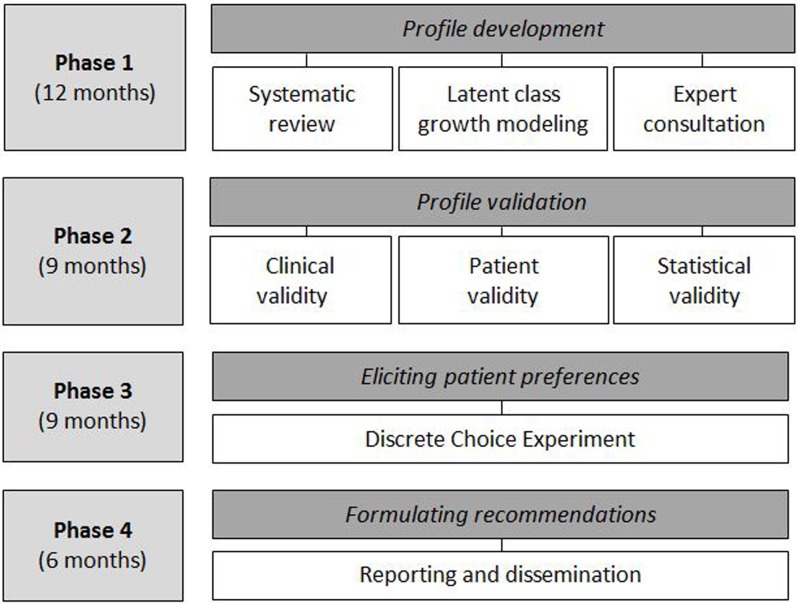
Study design and phasing.

PROFILe is designed as a practice-based, mixed-methods research comprising four phases, which are completed sequentially over a total period of 36 months. The project started in December 2014. Study design and phasing are shown in Figure 1. The research is conducted at Maastricht University in the Netherlands, in close collaboration with various stakeholders, and funded by Novo Nordisk. No ethical approval is needed for the research: as the data used are already available and patients are not physically involved in the research, the study is not subject to the Dutch Medical Research (Human Subjects) Act (WMO). PROFILe draws in considerable part on the 10-year, epidemiological Maastricht Study [16], which has previously been approved by the medical ethical committee of Maastricht University Medical Centre (MUMC+) (NL31329.068.10) and the Netherlands Health Council under the Dutch Population Screening Act (Permit 131088-105234-PG).

### Setting

Over the past decade, diabetes has become a public health priority for the Dutch Ministry of Health [17]. Considerable resources have been and still are invested in reforming the content, organization and funding of diabetes management with the aim of improving care quality and outcomes for patients. According to Wensing et al. [18], the Dutch Ministry of Health regards diabetes as ‘an ideal case for general policies for chronic illness care’. Indeed, some of the most important changes of late in Dutch chronic care management have started with pilots in diabetes care and were consequently rolled out to, for example, COPD care and vascular risk management [19]. Internationally, the Netherlands is regarded as a pioneer of high-quality diabetes care, ranking second after Sweden on the 2014 Euro Diabetes Index which compared diabetes management in 30 European countries [20].

In the Netherlands, the vast majority (85–90%) of patients with type 2 diabetes are managed by GPs in primary care [21]. Patients who need more complex management are treated in secondary care by a diabetes team led by an endocrinologist. According to the National Transmural Agreement (NTA) for type 2 diabetes [22], complex management concerns patients ‘who are unable to reach individual treatment targets in primary care (and for whom there are valid grounds for expecting improvement in secondary care) and/or whose management is problematic due to severe complications or therapy resistant cardiovascular risk factors’. When patients are referred to secondary care, the endocrinologist assumes responsibility for their diabetes care, either indefinitely or until they can transition back to general practice. The NTA specifies the formal criteria for referrals between primary and secondary care [22].

Because primary care is widely considered to be the most suitable medical home for chronically ill [23], and most Dutch type 2 diabetes patients are treated there, PROFILe will develop patient profiles specifically for the primary care setting. In recent years, Dutch primary care has undergone a considerable transformation as most GPs have gathered in so-called ‘care groups’. These provider networks are similar to Accountable Care Organizations in the United States and Clinical Commissioning Groups in the United Kingdom [24,25]. Care groups first emerged in Dutch primary care in 2007 with the experimental introduction of a bundled payment system for integrated type 2 diabetes care. Quickly growing in number, there are now around 100 groups covering near to all Dutch regions and 85 to 90 percent of type 2 diabetes patients [26]. Annually, care groups negotiate a bundled payment contract with health insurers to organise, coordinate and provide the whole package of non-complex type 2 diabetes care for patients in their region. The care group is responsible for all patients covered by its bundled payment contract; GPs (and affiliated personnel, such as practice nurses) deliver care themselves and/or subcontract services from other providers, such as physical therapists, dieticians, laboratories, and, to a limited extent, medical specialists. The content of the care package is prescribed by a national standard for diabetes care developed by the Dutch Diabetes Federation, which stipulates, amongst others, that patients are seen in general practice at least four times annually, receive a specific number of tests and screening, and are offered education about their disease and self-management [24].

Although diabetes care in the Netherlands is viewed internationally as ‘best practice’, recent evaluations suggest there is room for further improvement. Most notably, the role that patients have in their care remains limited, with support interventions for self-management still largely in their infancy [11,19]. Another limitation is the high level of service standardisation based on the Dutch diabetes care standard, which – according to the Euro Diabetes Index – is followed ‘so strictly that new ideas not accepted in the standard are shunned’ [20].

### Conceptual framework

Aim of the PROFILe project is to develop and validate a robust and feasible set of patient profiles that can be used in daily practice to support more patient-centered, tailored chronic care management. Although in essence, the patient profiles to be developed constitute a tool for case-mix classification – for which many other methods exist that have been studied extensively over the past years [28,29] – they will be unique in combining both (bio)medical patient features, such as disease duration and severity, and non-(bio)medical patient characteristics, like age, sex and educational level. Using non-(bio)medical characteristics for stratification purposes is assumed to provide better insight into patients’ abilities for self-management of their chronic condition(s) and, in so doing, enables the intensity of professional-led care to be matched optimally to patients’ actual care needs.

**Figure 2:**
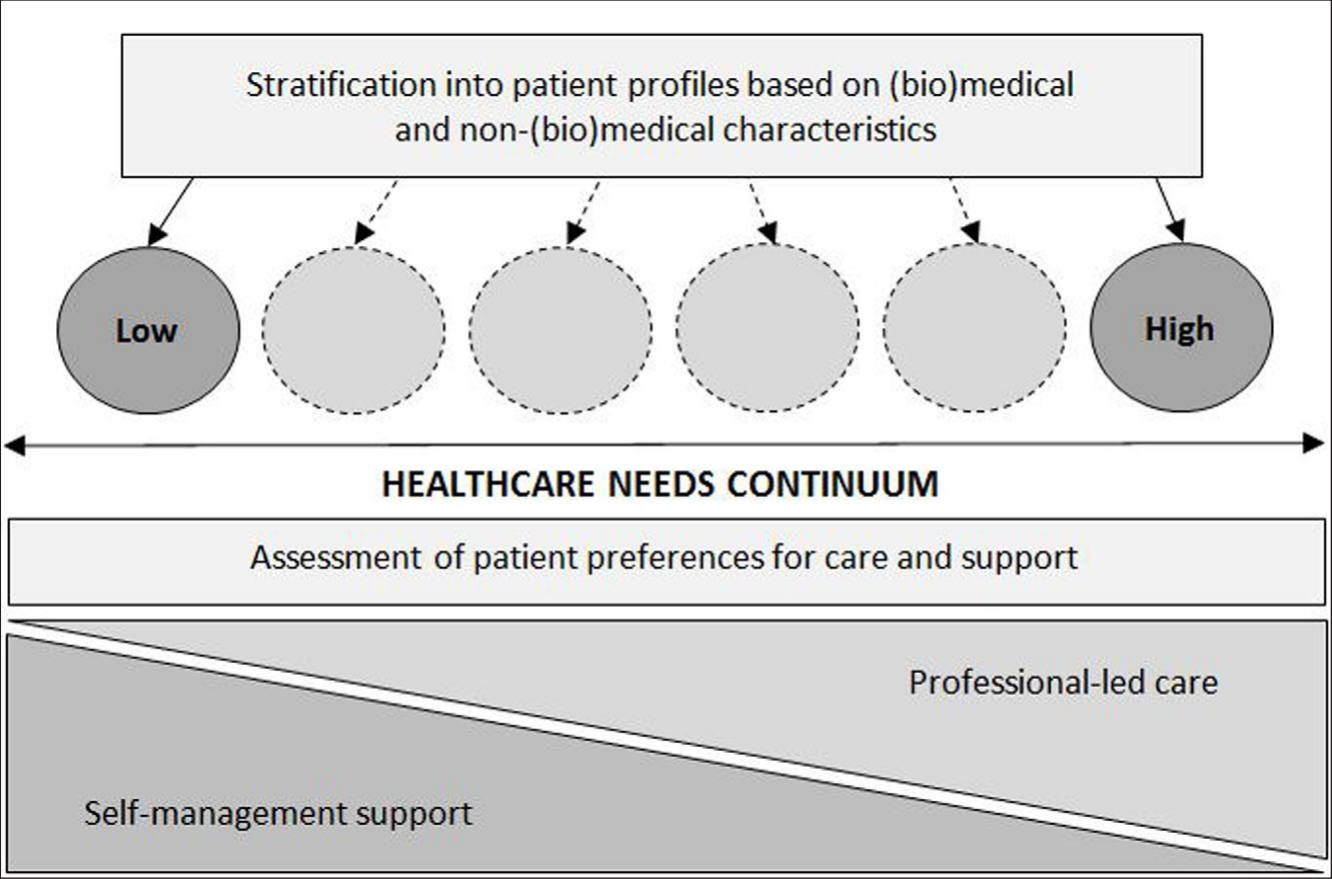
Framework for tailored chronic care management based on patient profiles.

Figure 2 shows the conceptual framework underlying PROFILe, which draws upon the Population Health Conceptual Framework of the Care Continuum Alliance [30]. The figure illustrates that the ultimate goal of profiling is to enable patient subgroups to be aligned with interventions across the continuum of self-management support and professional-led care that match their established level of healthcare needs as well as their preferences for specific services. Thus, patients with a low level of healthcare needs – based on their (bio)medical and non-(bio)medical characteristics – might prefer support by a community nurse and/or incidental email contact with a primary care provider to manage their health. On the other end of the spectrum, those with a high-needs profile could favour regular monitoring in general practice combined with individual, nurse-led education. However, rather than assuming patients’ likings for specific configurations of care and support, the PROFILe project will utilise a research method called ‘discrete choice experimentation’ to gain insight into the actual preferences of chronically ill patients for various attributes of chronic care management, such as the frequency of professional monitoring, central care giver, and methods and tools for self-management support. Moreover, as patients’ perception of their illness is known to often differ from health professionals’ assessment, the validity of the profiles will be tested against patients’ own perceptions of their level of healthcare needs.

### Data collection and analyses

The PROFILe project will combine a mixture of quantitative and qualitative data and analytic methods across four research phases.

#### Phase 1: Profile development

During the first research phase (12 months), the objective is to draft a robust and feasible set of patient profiles for tailoring type 2 diabetes management. Three research methods will be used to identify key patient characteristics influencing diabetes control and subsequently combine those factors into real-valued prediction models: (a) systematic literature review; (b) latent class growth modeling; and (c) expert collaboration.

##### Systematic literature review

The systematic literature review is intended to gain insight into which bio(medical) and non-(bio)medical variables are potentially relevant for assessing the healthcare needs of type 2 diabetes patients. For this purpose, we will synthesise existing evidence about characteristics of patients that cause heterogeneity in the utilization and clinical outcomes of disease management strategies. In line with previous research [5,6,7,8], ‘disease management’ is operationalised as interventions targeting at least two of the four practice-level elements of the Chronic Care Model, that is, self-management support, delivery system design, decision support and clinical information [31].

Searches for English language empirical studies published between 1998 and 2015 will be conducted in PubMed, EMBASE and CINAHL using multiple groups of search terms related to type 2 diabetes, disease management, the Chronic Care Model, patient characteristics and relevant outcomes. The latter will include various measures of diabetes control and resource utilization. Included articles will be analysed descriptively; in addition, the two to three most consistently reported outcome variables across included articles will be meta-analysed to explain heterogeneity in disease management outcomes based on variation in patient characteristics.

##### Latent class growth modelling

In the second part of the profile development phase, quantitative data analyses will be conducted using a technique called latent class growth modelling (LCGM). LCGM is a type of cluster analysis that is increasingly employed in clinical research to capture heterogeneity between individuals in, for instance, treatment responses or disease patterns [32]. Using LCGM, subgroups of patients with distinct clinical trajectories over time can be identified and their characteristics determined [33].

Within PROFILe, LCGM will be applied to identify classes of type 2 diabetes patients with unique trajectories over the course of time in three measures of diabetes control, that is, HbA1c, LDL cholesterol and systolic blood pressure, as well as in a composite of these three measures. Longitudinal data on these and other relevant measures are collected from the Diabetes Patient Registry of the regional care group in Maastricht, which has been providing integrated type 2 diabetes care based on bundled payment contracts since 2007. Based on its achievements, the group was recently designated one of nine ‘pioneer sites’ in population (health) management in the Netherlands by the Minister of Health [34].

The Diabetes Patient Registry contains individual patient data registered during primary care visits from 2007 onward concerning a wide range of variables related to patient demographics, clinical status, and type and frequency of care provision. The study population will include all patients who entered the Diabetes Patient Registry at some point in time between January 2009 and December 2014 (N = ~9,000). Based on the Diabetes Patient Registry data, models with increasing numbers of classes will be run. Model fit and parsimony are assessed using the Bayesian Information Criterion and Lo-Mendell-Rubin Likelihood Ratio Test [32]. A standardised entropy score is calculated to determine the amount of ambiguity in class allocation [35]. Potential associations between various patient characteristics on the one hand and membership of a given class on the other will be explored using multinomial logistic backward regression analyses. All available determinants in the Diabetes Patient Registry will be analysed separately; correlations are assessed to test for co-linearity. Those determinants achieving a p-value <0.10 will be included simultaneously through a backward elimination method, resulting in a model that includes only significant (p < 0.05) determinants.

In addition, multinomial logistic backward regression analyses will be conducted for a subsample of Diabetes Patient Registry patients, that is, those patients participating in the Maastricht Study [16]. This detailed epidemiological study, which started in 2010, focuses on the etiology and pathophysiology of type 2 diabetes, its classic complications (i.e. cardiovascular disease, nephropathy, neuropathy and retinopathy), and its emerging comorbidities, including cognitive decline, depression, and gastrointestinal, respiratory and musculoskeletal diseases [16]. During three to four 4-hour visits per participant, state-of-the-art imaging techniques and extensive biobanking are used to determine health status in a population-based cohort of 10,000 individuals enriched with type 2 diabetes patients. The latter are recruited from the Diabetes Patient Registry of the regional care group in Maastricht. An in-depth description of the design of the Maastricht Study can be found elsewhere [16]. Included in the multinomial logistic regression analyses are Maastricht Study participants with at least 24 months of registered data in the Diabetes Patient Registry prior to their inclusion in the Maastricht Study (N = ~1,000), enabling combination of cross-sectional (Maastricht Study) data and longitudinal (Diabetes Patient Registry) data on the individual patient level. Compared to the Diabetes Patient Registry, the Maastricht Study adds extensive phenotype data as well as information on quality of life, lifestyle, socioeconomic and psychological features. These data will be used to place the latent classes developed based on the Diabetes Patient Registry data in a larger system of variables that may include hypothesised predictors not available in the Diabetes Patient Registry (e.g. education level) as well as potential long-term outcomes of latent class membership (e.g. quality of life) [36].

##### Expert and stakeholder consultation

Based on the combined findings from the literature review and LCGM analyses, a preliminary set of patient profiles is drafted by the research team in close collaboration with various stakeholders and scientific experts. These are represented in the project’s Stakeholder Group, which includes representatives from patient organisations, provider associations, health insurers and policymakers, and the Scientific Advisory Board gathering (inter)nationally renowned experts in type 2 diabetes, disease management, case-mix classification and risk stratification. A priori, we assume phase 1 to result in three to eight draft patient profiles which, based on a limited number of pertinent (bio)medical and non-(bio)medical variables, describe relatively homogeneous classes of chronically ill in terms of their healthcare needs.

#### Phase 2: Profile validation

During phase 2 of the research (9 months), the aim is to validate the draft patient profiles focusing specifically on clinical validity, patient validity and statistical validity.

##### Clinical validity

To assess clinical (i.e. face) validity, that is, the extent to which health professionals consider the draft profiles as valid for assessing patients’ healthcare needs, an electronic Delphi panel will be conducted with representatives of provider associations involved in type 2 diabetes management in the Netherlands. Relevant associations are the Dutch General Diabetes General Practitioners Advice Group (DiHAG), Diabetes and Nutrition Organization (DNO), Professional Organisation for Diabetes Care Providers (EADV), Diabetes Education Study Group (DESG), Royal Dutch Pharmacists Association (KNMP), Royal Dutch Society for Physical Therapy (KNGF) and the Dutch Internists’ Association (NIV). The aim is to include two representatives from each Dutch association involved in structured diabetes management, so as to compose a balanced Delphi panel with sufficient professional expertise and mixed backgrounds.

The RAND/UCLA appropriateness method [37] will be used to design multiple Delphi rounds, including: (a) an online survey to assess experts preliminary scores of the profiles in terms of validity; (b) a face-to-face expert meeting to discuss individual scores and, where necessary and possible, increase group consensus; and (c) individual reassessment on a paper-based survey to produce final scores. Additional rounds may be added if insufficient consensus is reached after the face-to-face meeting. The focus of the Delphi study will be on the validity – according to healthcare professionals – of each separate patient characteristic identified as relevant during the first research phase, as well as on the validity of different combinations of these characteristics into patient profiles.

##### Patient validity

Given that patient profiles are intended to support more patient-centered management of type 2 diabetes, validation of the profiles by patients is also considered crucial. We will use a mixed-methods approach to test the validity of the draft profiles against patients’ own views of their level of healthcare needs. The latter will be measured using the validated Problem Areas in Diabetes (PAID) questionnaire, which is a widely used, 20-item measure of emotional adjustment to life with diabetes [38]. A purposive sample of five to ten type 2 diabetes patients per draft patient profile will be selected from GP practices in Maastricht to participate in the profile validation.

The results of the PAID questionnaire form the input for an individual, in-depth follow-up interview, which aims to: (1) elaborate on patients’ PAID scores by providing them the opportunity to tell their illness narratives; and (2) compare patients’ own view of their level of healthcare needs with the profile chosen by the researchers. As the primary focus of patient validation is on the subjective experience of healthcare needs by the person who is chronically ill, a descriptive phenomenological approach is used for the interviews and analysis. Phenomenology requires researchers to look at things in a new way without predispositions and prejudices, thus enabling fresh, rich and new understandings of existing phenomena [39]. A semi-structured interview guide will be used during the interviews to steer the conversation; the number and nature of questions can vary depending on the respondent’s illness narrative. All interviews are audio-recorded. Data analysis will be conducted conform the descriptive phenomenological method using Hycner’s 15-step framework [40], which starts with individual interview transcription and ultimately results in a composite summary of all interviews capturing the essence of the phenomenon under study as experienced by respondents.

##### Statistical validity

Finally, the statistical validity of the draft patient profiles – in particular, their generalisability to other settings – will be tested using quantitative data collected retrospectively from a different, larger cohort of patients than the one used for developing the profiles. This cohort will comprise a comprehensive selection of type 2 diabetes patients from the three remaining primary care groups in the Dutch province of Limburg (besides the one in Maastricht). Limburg is chosen as validation site because of its relatively poor population health compared to other provinces in the Netherlands, especially in terms of chronic disease prevalence [41].

Together, the three selected care groups cover an estimated population of approximately 65,000 to 70,000 individuals with type 2 diabetes. The groups’ Diabetes Patient Registries will be used as source of retrospective data collection. Relevant parameters are identical to those used in research phase 1, that is, all routinely registered measures of patient demographics, clinical status, and type and frequency of care provision. Included in the validation sample are all adult (≥18 years) type 2 diabetes patients with at least 24 months of Diabetes Patient Registry data.

The generalisability of the draft profiles will be determined by assessing to which extent: (a) they cover the entire type 2 diabetes patient population in Limburg; (b) routine Diabetes Patient Registry data are sufficient to enable stratification into profiles and/or which additional data collection is necessary; and (c) identified trajectories and associations between patient characteristics and class membership are comparable. Based on the results of this research phase, the patient profiles will be adapted where necessary and finalised.

#### Phase 3: Eliciting patient preferences

The objective of the third PROFILe phase (9 months) is to provide insight into the patient preferences that exist per profile for specific configurations of diabetes care and support. For this purpose, a discrete choice experiment (DCE) will be conducted. Discrete choice experimentation is a validated, systematic approach for eliciting preferences, which has a strong theoretical basis in economic science and is increasingly used in international health systems to involve patients in health policymaking [42]. The technique is based on two assumptions: (a) that healthcare services can be described by their attributes; and (b) that an individual’s valuation depends on the levels of these attributes. When determining an optimal way to provide a service, such as tailored type 2 diabetes management, a DCE can be used to show how people are willing to trade between attributes.

The DCE to be conducted in this study will consist of five steps (see Table 1). First, five focus group discussions are held with purposive samples of four to eight type 2 diabetes patients per session. In selecting participants, we will ensure that each draft profile is represented by at least one person during each focus group discussion. Goal of the sessions is to select healthcare service attributes for inclusion in the DCE. Nominal group technique (NTG) will be used to prioritise attributes based on patients’ preferences [43], with preliminary identification of potentially relevant attributes based on two sources: (1) the Dutch Diabetes Federation’s care standard for type 2 diabetes [27]; and (2) the Dutch version of the Patient Assessment of Care for Chronic Conditions (PACIC) survey [44,45]. Examples of relevant attributes may include the frequency of professional monitoring, setting of care, involved providers, different methods and tools for self-management support, use of electronic applications, and so on.

**Table 1:** Steps of the discrete choice experiment (DCE) process and methods and sample size per step.

	DCE step	Method	Sample size

1.	Attribute identification and selection	Focus group discussions (N = 5) using the nominal group technique	4–8 respondents per focus group
2.	Assigning levels to the attributes	Based on existing evidence (e.g. guidelines, protocols)	–
3.	Developing scenarios	Based on chosen attributes and levels	–
4.	Establishing preferences	Patient survey	50 respondents per profile
5.	Data analysis	Regression analyses	50 respondents per profile

Second, levels are assigned to each of the identified attributes: the attribute ‘frequency of monitoring’, for instance, might have four levels (e.g. two, four, six or eight times per year). Third, scenarios are drawn up describing all possible service (or outcome) configurations given the attributes and levels chosen. For example, we could ask respondents to choose between these two scenarios: (a) to have four annual check-ups, with the nurse as central care giver; or (b) to have two annual check-ups, with the GP as central care giver. The number of scenarios to be developed will depend on the number of attributes and levels chosen.

Fourth, a patient survey is conducted to elicit patients’ preferences for the developed scenarios. Although there is limited guidance on sample size calculations for DCE patient surveys, Pearmain et al. [46] suggest that sample sizes over 100 are a proficient basis for modeling preference data. Within this study, we aim for a larger sample size and will include at least 50 respondents per draft profile. Thus, if the analyses in phases 1 and 2 result in a final set of six profiles, 300 patients will be needed to participate in the survey. Fifth, regression techniques are used to analyse patients’ survey responses in general as well as focusing specifically on the level of heterogeneity in results between profiles.

The discrete choice experiment will be designed, conducted and analysed following published guidelines [42,47]. Respondents for the focus group sessions and survey will be selected from the Diabetes Patient Registry of the regional care group in Maastricht. Based on the findings from this research phase, recommendations will be formulated on how to tailor type 2 diabetes management to the developed and validated patient profiles. Moreover, the survey itself constitutes a project deliverable that can be used internationally to elicit patients’ preferences for chronic care management.

#### Phase 4: Formulating recommendations

Aim of the final PROFILe phase (6 months) is to integrate the results of the three previous phases and derive evidence-based recommendations on which (bio)medical and non-(bio)medical patient characteristics should guide tailored chronic care management and how these can be combined into a robust and feasible profiling instrument for everyday practice. Explorations of the generalisability of findings to other conditions than type 2 diabetes will be an important focus in this phase. Findings are reported back to key stakeholders and disseminated to broader audiences in a variety of ways, including through scientific publications and conference contributions.

## Discussion

This paper describes the design of the PROFILe project (2014–2017), a practice-based, mixed-methods research aiming to develop and validate a robust and feasible set of patient profiles for tailored chronic care management. It builds upon findings from the European collaborative DISMEVAL project, which was conducted between 2009 and 2012, and showed, amongst others, that current chronic disease management approaches in Europe tend to be highly standardised, insufficiently patient-centered, and result in differential – and often less than optimal – treatment effects across populations of chronically ill [48,49].

There is increasing consensus that better chronic care management requires a more patient-centered, tailored approach [10], which combines the advantages of maintaining a certain level of standardisation with the benefits of increased individualisation and patient participation. In business terms, this might be referred to as mass customisation, which is a service delivery trend adopted by major international companies, such as Levi’s, Starbucks and Burger King. Mass customisation combines the flexibility and personalisation of custom-made service delivery with the low unit costs of mass production. In practical terms, the strategy is not about promising customers anything, anytime, anywhere and anyhow, but rather about differentiating services within a predetermined ‘envelope of variety’ ascertained from the client perspective [50].

PROFILe aims to support exactly such differentiation in chronic care management: patient profiles are intended as an instrument to segment the chronically ill population into subgroups with similar healthcare needs for whom – based on insight into their preferences – a range of matching care and support options can be developed. In the long run, tailored management based on patient profiles offers considerable potential for achieving Berwick’s Triple Aim [51] of health system performance: (1) to improve patients’ experience of care, by stimulating explicit inclusion of their healthcare needs and preferences in treatment decisions; (2) to improve population health and quality of life, by aligning patients with appropriate levels of treatment and self-management support; and (3) to reduce the per capita cost of care, by minimizing the over-, under- and misuse of healthcare resources that results, amongst others, from overly standardised service provision and a lack of patient self-management. In this respect, the PROFILe project fits within a broader health policy trend seen in many European countries, in which governments are rearranging healthcare services based on population health needs, and non-complex healthcare tasks and responsibilities are increasingly transferred back to patients and their families, not in the least for cost containment purposes [52].

An important strength of the PROFILe project is its use of a mixed-methods approach, combining quantitative and qualitative data and study techniques within and across research phases. In particular when investigating complex, multicomponent interventions, a mixed-methods design is increasingly viewed as superior to more classic methodological approaches such as the randomised controlled trial [53]. Another strong point of the study is the involvement of patients in multiple study phases and the use of innovative methods, such as discrete choice experimentation, in order to produce robust and meaningful findings that emphasise the patient perspective. Although more research has been and is being conducted internationally concerning individualisation of type 2 diabetes management [54,55], PROFILe is unique in its use of variables of non-(bio)medical nature for tailoring purposes. Given the strong impact that patients’ personal circumstances have on their ability to self-manage and their level of treatment adherence [56], broadening the scope of individualisation beyond (bio)medical factors to also include demographic, socioeconomic and psychological aspects is a key forte of the PROFILe project.

There are also some limitations. Most notably, the disease-specific nature of the profiles to be developed – intended for patients with a primary diagnosis of type 2 diabetes – limits the generalisability of results and hampers development of a generic instrument for tailored chronic care management. However, there are two important arguments in favour of focusing on diabetes. First, because it is a priority health problem in the Netherlands, focusing on diabetes enables us to capitalise on the full potential that so-called ‘big data’ in electronic diabetes registries offer for personalising care [17]. Second, type 2 diabetes is widely considered to be a good model for chronic disease in general, in particular given its strong association with comorbidities [9,16], and is used as such in many countries’ health policymaking efforts in chronic care, including in the Netherlands [18,57]. Another limitation concerns the setting of the study in primary care, which leads to exclusion of the 10 to 15% most complex cases of type 2 diabetes – i.e. patients who are treated in secondary care in the Netherlands [21] – from our profiling efforts. Although the Dutch NTA for type 2 diabetes [22] seeks to ensure care continuity and safety during transitions between primary and secondary care, patients with complex type 2 diabetes might still benefit from a more tailored approach based on patient profiles. Hence, it is important to broaden the scope of future research efforts beyond primary care to include all patients with type 2 diabetes. A final limitation of the study is the lack of prospective evaluation of the effects of tailoring diabetes management based on patient profiles, for example in a randomised controlled trial, which is beyond the scope of this development and validation project. Following PROFILe, further research is necessary to gain detailed insight into the impact of tailored diabetes management on a range of measures related to the Triple Aim, including patient experience, population health and costs.

## Competing Interests

The study is funded by a grant from Novo Nordisk Netherlands.
